# Zeolite Lightweight Repair Renders: Effect of Binder Type on Properties and Salt Crystallization Resistance

**DOI:** 10.3390/ma14133760

**Published:** 2021-07-05

**Authors:** Milena Pavlíková, Adéla Kapicová, Adam Pivák, Martina Záleská, Michal Lojka, Ondřej Jankovský, Zbyšek Pavlík

**Affiliations:** 1 Department of Materials Engineering and Chemistry, Faculty of Civil Engineering, Czech Technical University in Prague, Thákurova 7, 166 29 Prague, Czech Republic; milena.pavlikova@fsv.cvut.cz (M.P.); adela.kapicova@fsv.cvut.cz (A.K.); adam.pivak@fsv.cvut.cz (A.P.); martina.zaleska@fsv.cvut.cz (M.Z.); 2 Department of Inorganic Chemistry, Faculty of Chemical Technology, University of Chemistry and Technology, Technická 5, 166 28 Prague, Czech Republic; michal.lojka@vscht.cz (M.L.); ondrej.jankovsky@vscht.cz (O.J.)

**Keywords:** lightweight repair mortars, zeolite, lime hydrate, natural hydraulic lime, cement-lime binder, mechanical resistance, hygrothermal performance, salt crystallization

## Abstract

Rendering mortars with lightweight zeolite aggregates were designed and tested. The effect of the type of binder used was also researched. For the hardened mortars, macrostructural parameters, mechanical characteristics, hygric and thermal properties were assessed. Specific attention was paid to the analysis of the salt crystallization resistance of the developed rendering mortars. Quartz sand was fully replaced in the composition of mortars with zeolite gave materials with low density, high porosity, sufficient mechanical strength, high water vapor permeability and high water absorption coefficient, which are technical parameters required for repair rendering mortars as prescribed in the WTA directive 2-9-04/D and EN 998-1. Moreover, the zeolite enhanced mortars exhibit good thermal insulation performance and high sorption capacity. The examined rendering mortars were found to be well durable against salt crystallization, which supports their applicability in salt-laden masonry. Based on the compatibility of the repair materials with those originally used, the lime and natural hydraulic lime zeolite mortars can be used as rendering mortars for the repair of historical and heritage buildings. The cement-lime zeolite render is applicable for repair purposes only in the case of the renewal of masonry in which Portland cement-based materials were originally used.

## 1. Introduction

Plastering and rendering mortars have been an important part of cultural heritage for thousands of years, providing a specific design that is typical for different eras, providing support for wall paintings and stuccoes, as well as protection of covered structures. The most common binders in historical renders and plasters were usually (aerial) lime-based; the presence of lime has been detected in plaster samples from different periods [1,2,3,4], occasionally in combination with gypsum [5] or pozzolanic materials [6,7,8]. Research into the hydraulicity of binders commenced at the end of the eighteenth century, and since then, binders such as natural hydraulic lime and Portland cement analogues have also been used. Since the end of the nineteenth century, cement has become the most widely used binding material for mortars [9]. However, lime-based mortars are still a prevailing material in the field of historic preservation due to their compatibility with historical lime-based structures [3,8].

Renders and plasters play multiple roles in the proper functioning of structures. Aside from their design and structural protection value, they can also provide additional thermal, acoustic, and air quality improvement [10]. Because of their role in multiple aspects of building performance, new wall coverings should always be properly realized, and old ones should be maintained in good condition, ensuring their function, and repaired in the case of damage. This study is specifically focused on repair mortars, their proper function, durability and thermal performance.

The good thermal quality of a structure is, in general, necessary in order to provide a healthy and energetically efficient indoor environment for its owners and users. On a global scale, these qualities are necessary to achieve global environmental targets, e.g., the set of goals set by the European Parliament and the European Council. In 2018, the target of reducing the energy consumption in the EU by 2030 by at least 32.5% with respect to the levels of 1990 was established as a part of long-term climate policies towards 2050, leading to accomplishment of sustainable energy supply, minimized greenhouse gas emissions, climate-neutral policy overall, etc. [11].

About a decade ago, buildings were responsible for about 40% of energy consumption in the European Union [12]. The energy used for heating and cooling created about 50% of the total energy consumption in buildings [13]. Because of the permanent growth of the building sector, a reduction of energy consumption of both old and new buildings is a key factor in accomplishing the mentioned environmental goals. This reduction can be achieved, e.g., by improving the thermal performance of walls. Lower thermal conductivity and improved radiative properties of wall layers [14] can minimize heat loss, and the use of cool coatings can reduce cooling costs, which are crucial in warm climates [15]. The thermal qualities of mortars are most important in the case of historic buildings, which cannot use additional inside or outside thermal insulation. Therefore, it is necessary to preserve and renovate mortars not only in the case of their deterioration and loss in order to maintain a good state of masonry and a decorative look, but also in cases where higher energy efficiency is needed in the structure.

In the case of the rendering mortar deterioration, which occurs over years due to variable factors, e.g., changing environmental and mechanical conditions, and structural dampness, either some conservation strategy [16] or substitution/replacement of old mortar with renovating mortar is needed. An appropriate substitution must be chosen according to the substrate, the type of environmental exposure, and the structure conditions. An important property of restoration mortars is the compatibility of their mechanical and physical-chemical performance with the specific properties of the historic materials.

The requirements on the exterior repair mortars applicable to damp and salty walls, which are the main concern of this study, are given, e.g., by the WTA 2-9-04/D recommendations [17]. Aside from the requirements indicated earlier in this work, renovation renders should provide mechanical and freeze–thaw resistance, high vapor permeability, low capillary water absorption, and protection against water penetration [7], as their decay is often related to the water presence in the structure and therefore freeze–thaw cycles, salt crystallization, internal stresses, etc. The hydrophobicity of external restoration renders is also often ensured, together with a proper porous system [18]. On the other hand, some authors have reported that masonry and render damage is accelerated by the application of hydrophobized repair renders [19]. Typical consequences of the hydrophobic treatment of repair renders applied on masonry include a worsening of dampness, moisture and salt solution moving up in the wall, salt-induced spalling and flacking, increased moisture content related to the salt hygroscopicity, which shifts the sorption capacity of porous building materials to higher level, lower evaporation flow, etc. [20,21,22].

For restoration purposes, the use of lime-, natural hydraulic lime-, cement-lime-, and pozzolanic-lime-based mortars, e.g., lime-metakaolin-based mortars, has been considered, with the majority considering the use of lime-based mortars [23,24,25,26,27,28,29,30,31,32,33]. In any case, in construction practice, despite compatibility problems, lime-cement repair mortars are the most commonly used in commercial materials for the repair and rendering of masonry structures. 

The properties of rendering mortars may be modified not only by the choice of binder, but also by, e.g., differences in the additives and aggregates used. As about two-thirds of the mortar volume is occupied by aggregate, the choice of aggregate type significantly affects the final render performance. Various studies have focused on the use of lightweight aggregates. These aggregates are suitable for renovating renders, as their porous structure helps renders to achieve higher porosity. Such structures are necessary to accommodate salt crystallization products and to avoid the deterioration of render caused by them and by freeze–thaw cycles. Due this fact, the use of, e.g., crushed lava [34] pumice [35], activated carbon [10], silica gel [10], ceramic waste [36,37], and perlite [30] in renovating renders has been suggested. These renders have been recognized as suitable due to their ability to accumulate considerable amounts of salts within their porous system [38]. Another advantage of lightweight aggregates in mortars, connected to their high porosity, is a positive influence on thermal insulation performance [30]. 

According to recent studies, zeolite has shown multiple advantages when used in rendering and repair mortars. Zeolites have been used in mortars either as a partial substituent for cementitious materials [39,40] or lime [32,41], as a mineral pozzolanic admixture [42] due to its pozzolanic nature, and as an aggregate [10,18,35,38]. 

Based on an analysis of recently published papers, new types of lightweight rendering mortars for the renovation of historical monuments have been developed. Natural zeolite has been used as a substitution for sand in the composition of lime-, natural hydraulic lime-, and cement-lime-based rendering mortars. The motivation of the research was to develop lightweight, porous, vapor-permeable, low-thermal-conductivity and salt-resistant mortars that cannot be produced solely with silica sand aggregate. A complex analysis of the effect of zeolite aggregate on the properties and durability of renders is provided. Additionally, the effect of binder type on the technical and functional parameters of the developed mortars was researched. A broad experimental campaign was carried out, comprising macrostructural analyses, assessment of mechanical parameters, testing of hygrothermal performance, and assessment of salt resistance, which was evaluated based on the conducted salt crystallization test. Such unique and comprehensive analysis, which also includes time-consuming tests of the combined effect of the binder type and zeolite on the performance and properties of mortars intended to find use in repair of historical masonry has not been presented yet, and represents another step in the development of advanced multi-functional repair mortars for construction practice. 

## 2. Materials and Methods

### 2.1. Composition of Examined Renders and Base Materials

Rendering mortars were prepared from hydrated lime CL 90-S delivered by the lime plant Čertovy Schody, Inc., Tmaň, Czech Republic, natural hydraulic lime NHL 3.5 produced by Zement- und Kalkwerke Otterbein GmbH & Co. KG, Otterbein, Germany, and Portland cement type CEM I 42.5 R manufactured by Českomoravský cement, Inc., Radotín, Czech Republic. Washed quartz sand (fraction 0/2 mm) with loose bulk density 1673 kg·m^−3^ (Filtrační písky, Ltd., Chlum, Czech Republic) and zeolite fraction 0/2 mm and loose bulk density 1049 kg/m^3^ (Zeocem, Inc., Bystré, Slovak Republic) were used as fine aggregate. Quartz sand was a mixture of three fractions (0.0/0.5 mm, 0.5/1 mm, 1/2 mm) that were mixed in the mass ratio 1:1:1. Similarly, zeolite was mixed from three fractions, 0.063/0.5 mm, 0.5/1 mm, and 1/2 mm, respectively. The mixing ratio was 1:1:1 by mass. The binder-to-aggregate volume ratio was 1:1.15 and was similar for all studied mortars. This corresponds with the 1:4 weight ratio commonly used in preparation of lime and cement-lime rendering mortars in practice, and has also been well documented in historical masonry [43,44,45,46]. The dosage of mixing water was adjusted to maintain the normal consistency of fresh mortar with the flow value of (160 ± 5) mm, measured using the flow table test according to the standard EN 1015-3 [47]. The composition of prepared rendering mortars is introduced in Table 1. Reference mortars with quartz sand aggregate were labeled LM-R, NHLM-R, and CLM-R, where acronyms LM, NHLM, and CLM stand for lime mortar, natural hydraulic lime mortar and cement-lime mortar. In mortars LM-Z, NHLM-Z, and CLM-Z zeolite was fully replaced with sand while maintaining the binder-to-aggregate volume ratio unchanged. 

The fresh mortar mixtures were cast into steel molds, demolded after 48 h, and stored at temperature 23 ± 2 °C and high relative humidity 90 ± 5% until they were tested. The casted samples were 40 mm × 40 mm × 160 mm prisms, 100 mm cubes, and circular plates with radius of 120 mm and thickness of 30 mm. 

### 2.2. Analysis of Base Materials

Chemical composition of raw materials was determined by X-Ray Fluorescence (XRF) analysis (EDXRF Spektrometer, ARL QUANT’X, Thermo Scientific, Waltham, MA, USA). 

The diffraction pattern of zeolite was measured on a D8 Phaser powder diffractometer (Bruker, Karlsruhe, Germany) with parafocusing Bragg-Brentano geometry using CuKα radiation (*λ*—0.15418 nm, U = 30 kV, I = 10 mA).

Using a scanning electron microscopy (SEM) with a FEG electron source (Tescan Lyra dual beam microscope), the morphology of zeolite was investigated. Elemental composition and mapping were performed using an energy dispersive spectroscopy (EDS) analyzer (X-MaxN) with a 20 mm^2^ SDD detector (Oxford instruments, Abingdon-on-Thames, UK) and AZtecEnergy software. To conduct the measurements, the samples were placed on a carbon conductive tape. SEM and SEM-EDS measurements were carried out using a 10 kV electron beam and 10 mm working distance between sample and detector.

The particle size distribution curves of zeolite and sand mixtures were measured in the standard sieve analysis; the mesh size was 2 mm, 1 mm, 0.5 mm, 0.25 mm, 0.125, 0.063 mm, and 0.001 mm, respectively.

Microstructure of zeolite was characterized by MIP analysis, which was conducted by the set of two porosimeters of Pascal series, Pascal 140 and Pascal 440 (Thermo Fisher Scientific, Waltham, MA, USA). The measured parameters were total porosity, average pore diameter, and total cumulative pore volume. 

### 2.3. Testing of Hardened Rendering Mortars

The 28-day and 90-day samples were analyzed. A minimum of 5 samples was tested.

#### 2.3.1. Assessment of Macrostructural Parameters 

Among the macrostructural parameters, bulk density, specific density, and total open porosity were measured. The dry bulk density *ρ*_b_ (kg·m^−3^) was measured in compliance with the European standard EN 1015-10 [48]. The samples were vacuum dried (Vacucell, BMT, Brno, Czech Republic) at 60 °C until their equilibrium mass was achieved (difference in sample mass was <0.1%). The specific density *ρ*_s_ (kg·m^−3^) was determined using a helium pycnometer Pycnomatic ATC (Porotec, Hofheim, Germany). Based on the knowledge of the dry bulk density and specific density values, the total open porosity *ψ* (-) was calculated [49]. The expanded combined uncertainties of the bulk density, specific density, and porosity determination were 1.4%, 1.2%, and 2.0%.

#### 2.3.2. Determination of Mechanical Parameters 

Flexural strength, compressive strength, and dynamic modulus of elasticity were the investigated mechanical parameters. The strength tests were performed according to the standard EN 1015-11 [50]. In the three-point bending test, the flexural strength *f*_f_ (MPa) was measured on the 40 mm × 40 mm × 160 mm prisms. The applied load speed was 50 N·s^−1^. The compressive strength *f*_c_ (MPa) was measured on the fragments from the flexural strength testing. The uniaxial compression force (100 N·s^−1^) was applied on the 40 mm × 40 mm cross section of the specimens. The dynamic modulus of elasticity *E*_d_ (Gpa) was measured using a Vikasonic apparatus (Schleinbinger Geräte, Buchbach, Germany). The expanded combined uncertainties of the mechanical parameter assessments were 1.4%, 1.4%, and 2.3% for *f*_f_, *f*_c_, and *E*_d_, respectively.

#### 2.3.3. Water and Water Vapor Transport Properties

The water absorption coefficient *A*_w_ (kg·m^−2^·s^−1/2^) test was conducted in accordance with the EN 1015-18 [51]. The 40 mm cubes were insulated on lateral sides by epoxy resin and their bottom side was submerged 5 mm in water. The automatic balance allowed continuous recording of increasing sample mass. Based on the measured results, the water absorption coefficient was evaluated as described by Fang et al. [52]. Based on the water absorption coefficient, the apparent moisture diffusivity *κ*_app_ (m^2^·s^−1^) was calculated using the procedure formulated by Kumaran [53,54]. The expanded combined uncertainty of the water absorption tests was 1.2%, and that of apparent moisture diffusivity 2.9%.

The water vapor transmission properties were quantified in the wet-cup and dry-cup tests that were arranged and conducted according to the EN ISO 12572 [55]. The circular plate samples had diameter 120 mm and thickness 30 mm. The relative humidity ratio in the water vapor transmission test was 93/50 ± 5% in the wet-cup test arrangement and approximately 2/50 ± 5% in the case of dry-cup experiment. Saturated KNO_3_ solution and silica gel were used for the generation of required relative humidity in the cups. From the measured steady-state sample mass and gain, the water vapor resistance factor *μ* (-) was calculated. The expanded combined uncertainty of the water vapor resistance factor assessment was 2.8%.

#### 2.3.4. Thermal Properties

Heat transport and storage parameters of hardened samples were determined by a thermal constants analyzer ISOMET 2114 (Applied Precision, Bratislava, Slovakia) operating on a transient impulse technique principle [56]. For the measurement, a circular surface probe IPS 1105 was used. The measurement range of the applied probe was 0.04–0.3 W·m^−1^·K^−1^ for the thermal conductivity λ (W·m^−1^·K^−1^) and 4.0 × 10^4^–1.5 × 10^6^ J·m^−3^·K^−1^ for the volumetric heat capacity *c*_v_ (J·m^−3^·K^−1^). The measurement of thermal parameters was done in dependence on moisture content, from the dry to fully water saturated state. 

#### 2.3.5. Salt Crystallization Resistance

In the assessment of salt crystallization resistance of the investigated mortars, standard EN 12370 [57] was followed. To reflect real salinization of masonry materials, sodium chloride (NaCl) and sodium sulfate (anhydrous Na_2_SO_4_) were chosen as penetrating salts. As the EN 12370 [57] defines testing of salt crystallization resistance of natural stone, it overestimates salt concentration in the salt crystallization resistance test. With respect to the recommendations reported in [58,59], the concentration of each salt used was chosen to be 2% (weight salt/weight dry specimen). Oven-dried 90-day specimens having dimensions of 40 mm × 40 mm × 40 mm were subjected to 10 crystallization cycles; each cycle consisted of immersion of the samples in salt solution for 2 h followed by drying in an oven at 70 °C for at least 16 h. After drying, specimens were removed from the oven and left to cool for 2 h. Each sample was placed in its own container, which was covered with the cap to prevent evaporation during the immersion and cooling phase. For the evaluation of the salt crystallization effect, visual observation, light microscopy (LM) analysis, loss or gain of specimen mass after crystallization cycles, and porosity tests were performed. The light microscopy (LM) was performed with Navitar (Rochester, New York, NY, USA) macro-optics with optical zoom up to 110X and recorded with a digital camera Sony 2/3”, with a resolution of 5 Mpix. The porosity changes due to salt crystallization were obtained from the bulk density and specific density data, similar to the process explained above for testing the samples that were not subjected to the salt resistance test. In the measurement mass loss or gain, the samples exposed to the 10 wetting/drying cycles were leached at 200 mL of water. The leaching took 24 h at 80 °C.

#### 2.3.6. Measurement of Sorption and Desorption Isotherms

To evaluate the effect of salt presence on the hygroscopicity of the renders, sorption and desorption isotherms of prepared mortars were measured using a dynamic vapor sorption device DVS-Advantage II (Surface Measurement Systems, Alperton, London, UK). In the DVS test, dry samples that underwent exposure to the specific salt solution (with the salt crystallization test) or water and wetting/drying cycles were examined. The samples were exposed to the following partial relative humidity (partial pressure) profile: 0, 20, 40, 60, 80, and 98%. The temperature of the DVS tests was set to 20 °C. During the experiments, the DVS-Advantage instrument was running in *dm*/d*t* mode (the mass variation over the time variation) to decide when equilibrium was reached.

## 3. Results and Discussion

The chemical compositions of the base materials as determined by XRF analysis are given in Table 2. Only the main elements are given. Oxygen makes up the bulk of the rest of the sum of the elements, as most of the elements are present as oxides.

**Figure 1:**
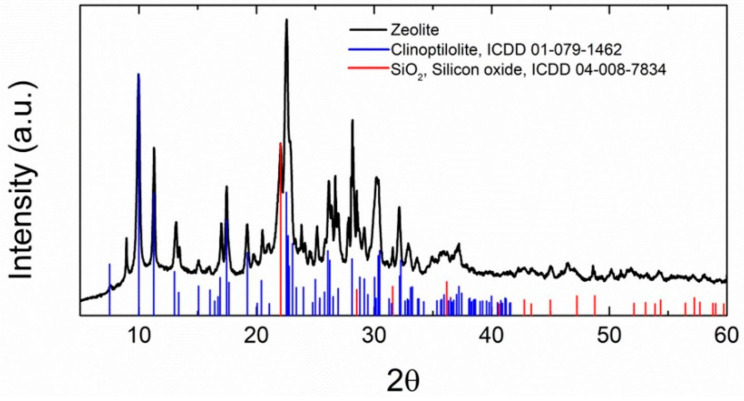
Diffraction pattern of zeolite.

The diffraction pattern of zeolite is presented in Figure 1. The identified crystalline structures corresponded to clinoptilolite (natural zeolite) and silicon oxide. The phase composition of the applied binders was reported recently in [34]. 

**Figure 2:**
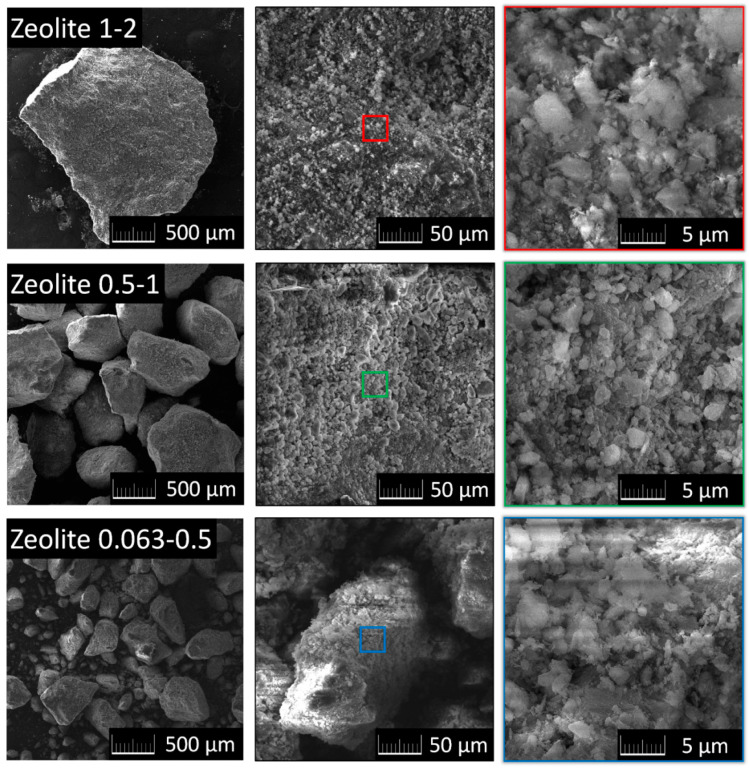
Microstructure of different zeolite fractions observed using SEM.

Different grain sizes of zeolite have no significant effect on the microstructure. The microstructure is typical for this type of mineral, where the resulting agglomerates are formed by particles with different shapes and sizes between 2 and 10 μm (Figure 2). The size of the fraction also does not affect the chemical composition, which was assessed by EDS analysis and is shown in Table 3. In addition to Si, Al, and O, which form the main structure of zeolite (approximately 90 wt.%), elements such as K, Ca, and Fe were also detected by EDS, which is in agreement with the XRF data. Let us note that a small amount of carbon was also detected, which originated from the conductive carbon tape used to conduct the measurements.

**Figure 3:**
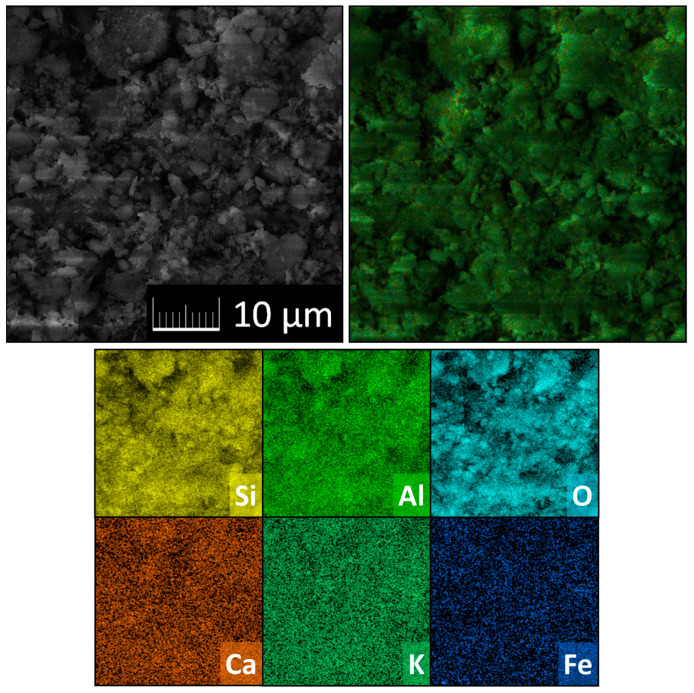
Elemental maps of zeolite, fraction 0.5–1 mm.

As the EDS elemental maps were highly comparable for all zeolite fractions, only maps for the fraction 0.5–1 mm are shown (Figure 3). The distribution of elements in the zeolite structure is homogeneous.

**Figure 4:**
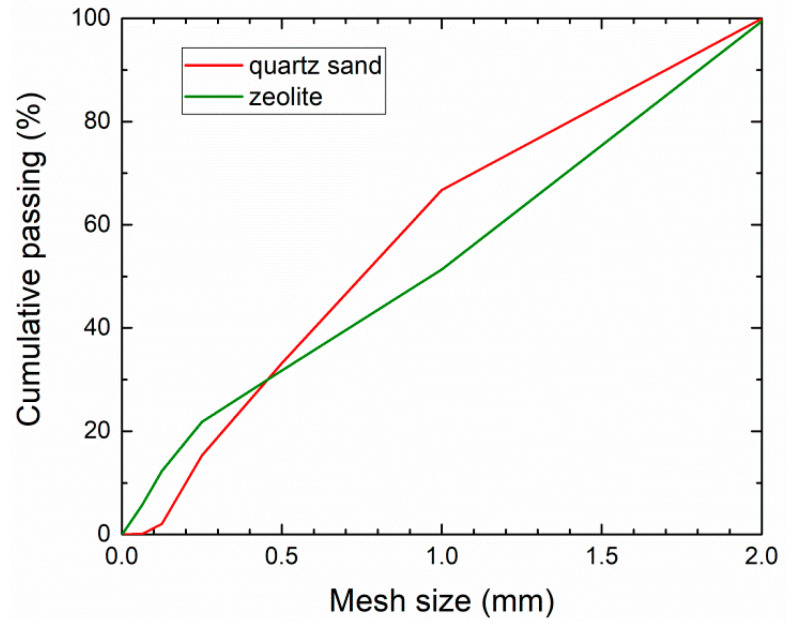
Particle size distribution of zeolite and quartz sand mixtures.

The particle size distribution curves of zeolite and sand mixtures measured by the standard sieve analysis are shown in Figure 4. 

The microstructure parameters of zeolite determined by MIP were as follows: total porosity 24.9%, average pore diameter 0.031 µm, and total cumulative pore volume 0.16 cm^3^·g^−1^. Compared to river sand, which is assumed to have negligible porosity, zeolite was found to be highly porous.

The results of structural, mechanical, hygric, and thermal properties were evaluated using the specifications for masonry and repair mortars summarized in the EN 998-1 [60] and the WTA directive 2-9-04/D [17]. 

The fundamental physical parameters of hardened rendering mortars are introduced in Table 4. The effect of the curing age of the tested samples is quite apparent. Typically, in comparison with 28-day renders, the 90-day matured samples exhibited higher bulk density and thus lower porosity. This was due to the continuous hydration, carbonation of lime binder and slow activation of reactive minerals in NHL-based mortars. The zeolite lightened renders yielded bulk density values well below 1400 kg·m^−3^, which is prescribed in the WTA directive 2-9-04/D [17]. Similarly, the porosity of repair renders must be >40% [17], which was met by all mortars with zeolite. Compared to the reference samples with quartz sand, the porosity of the zeolite-containing mortars was greatly increased, which supports their presumed application for repair purposes with respect to their vapor permeability and safe salt accumulation. The increase in porosity was mainly due to the high porosity of zeolite itself. 

The mechanical parameters of the investigated renders are presented in Table 5. The mechanical strength and stiffness are the result of three mutual effects: porosity, type of aggregate, and nature of the binder. The time development of mechanical parameters is clearly apparent for all examined materials. The highest strength and stiffness were exhibited by cement-lime mortar, which is ranked according to the EN 998-1 [60] in category CS III. Natural hydraulic lime mortars are classified into category CS II and lime mortars belong to CS I. As prescribed in EN 998-1 [60] and WTA directive 2-9-04/D [17], repair mortars must meet the criteria of the CS II class, i.e., the compressive strength of this type of mortar must be in the range 1.5–5.0 MPa. However, in the literature there are several examples where much lower compressive strength values than those prescribed by WTA are recommended for the repair or conservation of traditional air lime renders. In [27,61], the authors reported on the sufficient 90-day compressive strength in the range 0.4–2.5 MPa of repair mortars. In this respect, the rendering mortars LM-R and LM-Z can be considered as suitable materials for repair applications, especially in structures where lime renders were originally used. On the other hand, the mortars CLM-R and CLM-Z are too rigid, and are thus incompatible with the materials of historical masonry, and cannot be recommended for repair applications except in cases where cement-lime renders are being reconditioned. This is in agreement with the generally accepted position of heritage authorities and those working in the field of repair and conservation of old and historical buildings [62,63,64,65]. 

In the EN 998-1 [60] and WTA directive 2-9-04/D [17], no strict criterion for the modulus of elasticity is presented. In [66,67], the authors recommend a modulus of elasticity in the range 2–6 GPa for repair render, which safely meets the requirements of lime and natural hydraulic lime mortars. The stiffness of cement-lime mortars is too high to be applicable in restoration and repair works. 

The results of the wet-cup and dry-cup tests are presented in Table 6. 

The acceleration of water vapor transport in mortars with zeolite is obvious. Lime and natural hydraulic lime mortars have a water vapor resistance factor <12.0, which is strictly required by the WTA directive 2-9-04/D [17]. In this case, the criterion introduced in the WTA directive is much more limiting than that prescribed in EN 998-1 [63], which requires µ < 15.0. Based on the obtained data, LM and NHLM materials can be considered highly permeable for water vapor, which can help to dry out the moist masonry and improve the hygrothermal state of the repaired structure. A similarly high water vapor transmission rate was reported, e.g., by Silva et al. [68], who analyzed blended lime-hydraulic lime mortars. High water vapor permeability of lime mortars was acknowledged also by Parcesepe et al. [69]. The cement-lime mortars yielded higher resistance to water vapor transport, which limits their use on masonry suffering from excessive moisture content. On the other hand, these mortars can be still considered to be vapor permeable and can find use in the fabrication of new buildings or for the repair of cement-lime mortars when the risk of possible moisture-induced damage is unexpected.

The reduced surface bonding of water vapor molecules in the wet-cup experiment resulted in the acceleration of water vapor transmission, and thus lower water vapor resistance factor in comparison with the results of the dry-cup test. Such performance of porous building materials has been documented, e.g., in [70,71,72]. 

The water absorption coefficient *A*_w_ and the apparent moisture diffusivity *κ*_app_ data is shown in Table 7. Similarly to the case of water vapor transport, the use of zeolite in mortar composition resulted in increased water transport parameters, which were slightly reduced with prolonged sample hardening. This is in agreement with the porosity data. According to EN 998-1 [60], the repair mortar must have *A*_w_ ≥ 0.3 kg·m^−2^·s^−1/2^, which meets the requirements of the LM-Z and NHLM-Z rendering mortars. The water absorption coefficient of CLM-Z mortar is low and does not warrant the use of cement-lime mortars in repair applications. 

**Figure 5:**
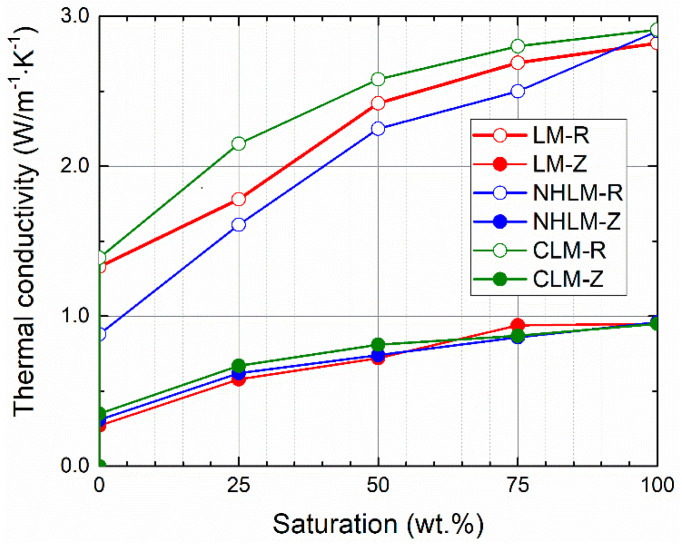
Moisture-dependent thermal conductivity—28-day samples.

**Figure 6:**
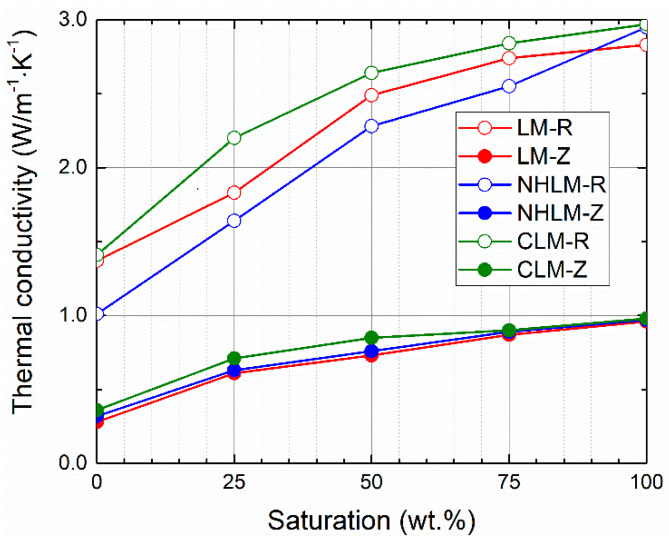
Moisture-dependent thermal conductivity—90-day samples.

**Figure 7:**
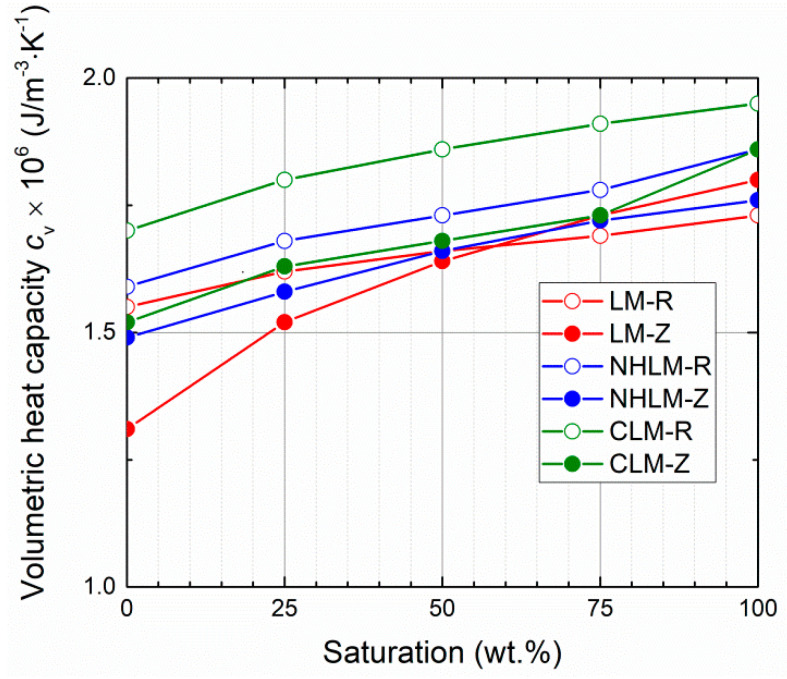
Moisture-dependent volumetric heat capacity—28-day samples.

Moisture-dependent thermal properties are graphed in Figure 5, Figure 6 and Figure 7. As only small differences in the resulting thermal parameters were observed between the 28-day and 90-day samples, only data on the thermal conductivity are presented for both curing ages. 

Moisture presence greatly increased both the thermal conductivity and volumetric heat capacity. This fact must be always considered when designing renovation treatment for historical masonry that usually suffers from excessive moisture presence and damage. The investigated thermal parameters were reduced by the use of zeolite instead of quartz sand in the mortar composition. This was due to the lower density of zeolite compared to that of quartz sand and the higher porosity of mortars with zeolite-based aggregate. The differences in the thermal conductivity were higher for materials with quartz sand, whereas the thermal conductivity/water saturation curves were for mortars with zeolite very close to each other. In the case of reference mortars, the lowest dry thermal conductivity values were for both curing ages found for natural hydraulic lime mortars, and the highest dry thermal conductivity was recorded for cement-lime mortars. Conversely, the lowest saturated thermal conductivity was measured for lime mortars. The volumetric capacities measured in the dry state of 28-day samples were the lowest for lime mortars, the highest for cement-lime mortar, and those of the natural hydraulic lime mortars were in between. Similarly to the case of thermal conductivity, the low density and unit weight and high porosity of zeolite, together with the higher porosity of mortars with zeolite, resulted in reduced volumetric heat capacity compared to the quartz sand mortars. 

From a technical point of view, there is no strict requirement with respect to the thermal performance or parameters of mortars intended for repair works. However, the dry thermal conductivity of mortars with zeolite was low (0.27-0.35 W·m^−1^·K^−1^), which could help to mitigate the heat losses through the masonry under repair and renovation.

Mass loss or gain after the salt crystallization test determined in accordance with the EN 12370 [57] is summarized in Table 8. The difference in porosity of laboratory-stored samples and those that underwent salt crystallization experiment is also presented. Due to the highly open porous structure, the differences in sample mass and porosity were small, which is evidence for the high resistance of the investigated rendering mortars against salt crystallization. The exposure of LM-R, NHLM-R, NHLM-Z mortars to salt action resulted in slightly decreased porosity, pointing to partial salt binding in the porous space of materials. On the other hand, the porosity of LM-R, CLM-R, and CLM-Z increased due to the salt action, which can be assigned to the partial damage of these materials. However, one must take into account that the differences in porosity were small, especially taking into account the measurement uncertainty, which was effected especially by mortar inhomogeneity and measured small differences in porosity values. However, as the recorded differences in porosity values were small, they clearly indicate the durability of the examined mortars with respect to salt-induced damage. 

**Figure 8:**
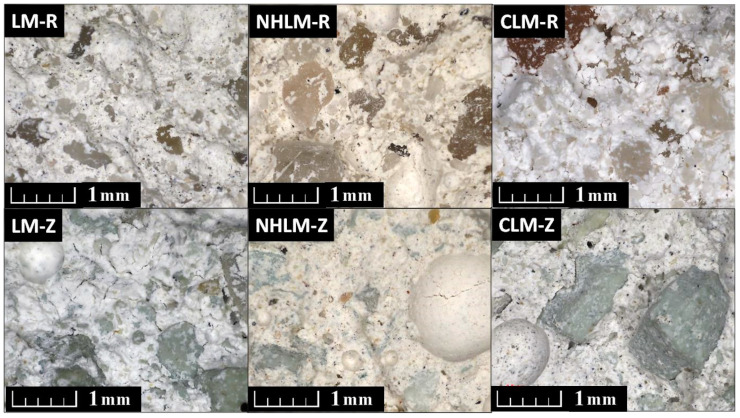
Structure of the renders exposed to the salt crystallization test.

The results of light microscopy imaging are presented in Figure 8. No cracks or any surface spalling, delamination and other damage types were observed on the plasters’ fracture surface, which clearly indicates the high salt crystallization resistance of the examined rendering mortars.

**Figure 9:**
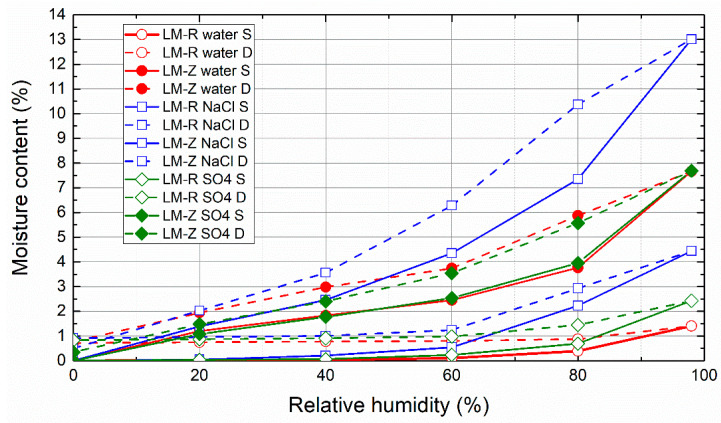
Sorption and desorption isotherms of lime mortars (LM).

**Figure 10:**
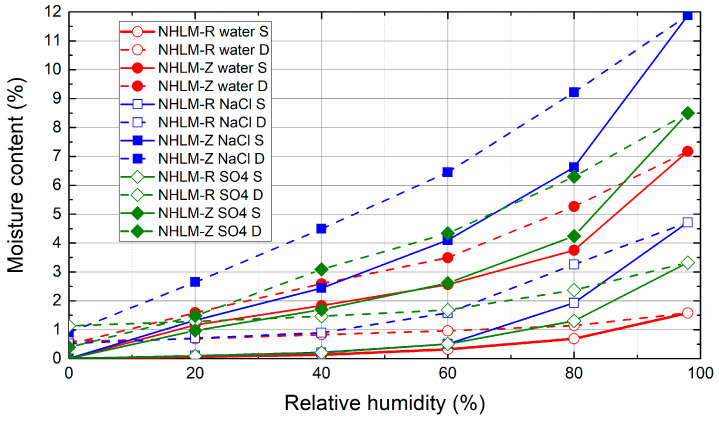
Sorption and desorption isotherms of natural hydraulic lime mortars (NHL).

**Figure 11:**
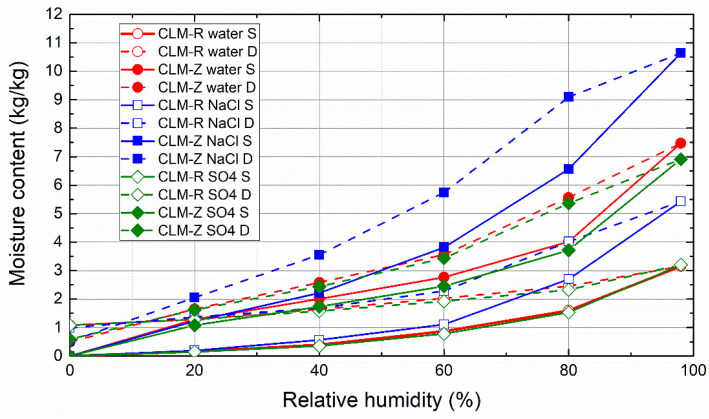
Sorption and desorption isotherms of cement-lime mortars (CLM).

The sorption and desorption isotherms of mortar samples that underwent the salt crystallization test and the controls exposed to cyclic wetting in pure water followed by drying are presented in Figure 9, Figure 10 and Figure 11. Independently of the binder type, the tendency to absorb water was greatly increased by the use of zeolite as an aggregate instead of sand. This was due to the higher porosity and thus highly open and free binding sites for water vapor molecules of zeolite-lightened mortars. For all of the studied mortars, the presence of NaCl resulted in significantly higher moisture content than that observed for control mortars that were not immersed in NaCl solution. Additionally, the presence of Na_2_SO_4_ in the porous space of the mortars intensified the water vapor adsorption, although the increase in the acquired moisture content was much lower than in the case of NaCl. This corresponds well with the deliquescence and crystallization or efflorescence relative humidity of NaCl and Na_2_SO_4_ published in the literature [73]. NaCl and Na_2_SO_4_ are reported to deliquesce at ≈75% and 84%, respectively, and effloresce at ≈43% and 57%, respectively [74,75]. This was the reason for the higher water vapor sorption capacity of mortars in the high relative humidity range. From a practical point of view of the design and planning of restoration treatments, the extreme hygroscopicity of NaCl must always be considered, especially in structures with relative humidity of the environment higher than NaCl deliquescence threshold. 

## 4. Conclusions

Rendering mortars lightened with zeolite aggregate were designed and tested. The effect of the type of binder used was also researched. Zeolite itself as a raw material was also characterized. The morphology, chemical composition and phase composition were determined in order to evaluate the suitability of this material for use as a substitute for sand in the composition of mortars. The broad experimental campaign was focused on the assessment of the macrostructural parameters, mechanical strength and stiffness, hygrothermal performance, and salt crystallization resistance of the developed mortars. Based on the tests conducted and the results obtained, the following main findings can be concluded, although one must take into account the fact that the obtained results were achieved for a specific type of zeolite with parameters as summarized below:

(i) The main elements identified in the alternative aggregate (zeolite) were Si and Al, which corresponded well with the recorded crystalline phases, i.e., clinoptilolite and silicon oxide. The agglomerates of zeolite exhibited different shapes, and sizes between 2 and 10 μm, and the size of particles had no effect on their chemical composition. According to XRF and EDS data, in addition to Si, Al, and O, elements such as K, Ca, Fe and Mg were observed. The chemical composition, microstructure and particle size of zeolite meet the prerequisites for its use as a silica sand replacement well.
(ii) The use of zeolite makes it possible to develop mortars whose bulk density and porosity conform to the requirements of WTA directive 2-9-4/D for repair rendering mortars.
(iii) In terms of the mechanical performance, lime and natural hydraulic lime mortars with zeolite are considered to be suitable for repair applications, especially in heritage buildings or structures, where similar type of binder was originally used. On the other hand, lightweight cement-lime mortar was found to be too rigid and cannot be recommended for repair applications except for cases in which cement-lime renders are being renewed.
(iv) The replacement of quartz sand with zeolite greatly accelerated the transmission of water vapor through the investigated mortars. The criterion set by WTA directive 2-9-4/D with respect to the water vapor permeability of repair mortars was easily met by lime and natural hydraulic lime mortars. Conversely, the water vapor resistance factor of cement-lime mortars was higher, thus limiting their use in damp masonry.
(v) Water transport was enhanced by the use of zeolite as an aggregate, and similarly to the case water vapor transmission, lime and natural hydraulic lime zeolite mortars met the criterion imposed by the EN 998-1 for repair mortars.
(vi) Due to the lower density of zeolite compared to quartz sand and the higher porosity of lightweight zeolite renders, both the thermal conductivity and volumetric heat capacity were significantly decreased. These mortars, when applied in the form of renders, can thus contribute to the mitigation of heat transport through the masonry under renovation.
(vii) The tested rendering mortars exhibited high resistance to the crystallization of NaCl and Na_2_SO_4_ solutions, as well as the ability to accommodate salts in their highly porous structure.
(viii) The use of zeolite as an aggregate greatly intensified the water vapor adsorption capacity of the tested renders, independently of the binder type.

Summarizing the findings highlighted above, it can be concluded that the developed lightweight zeolite renders with lime and natural hydraulic lime binder can be classified as repair rendering materials, and possess sufficient mechanical strength, high permeability for water and water vapor, provide a thermal insulation function, and offer great resistance against salt crystallization. Due to their compatible composition, they are well applicable for the repair of old and historical masonry, even in cases where salt and moisture are present. One must take into account the high sorption capacity of mortars with zeolite, which can be significantly further enhanced as a result of the presence of inorganic salts coming from salt-laden masonry and other sources, such as underground water.

The obtained data not only characterize the developed rendering mortars, but can be further exploited as input parameters for the computational modeling of coupled moisture, heat and salt in similar types of highly porous building materials. 

## Figures and Tables

**Table 1:** Composition of the investigated rendering mortars, kg·m^−3^.

Mortar	Lime Hydrate	NHL	Portland Cement	Sand 0.0/0.5	Sand 0.5/1.0	Sand 1.0–2.0	Zeolite 0.063–0.5	Zeolite 0.5–1.0	Zeolite 1.0–2.0	Water
LM-R	326.1	-	-	434.5	434.5	434.5	-	-	-	391.3
LM-Z	332.3	-	-	-	-	-	272.5	272.5	272.5	508.6
NHLM-R	-	410.0	-	464.9	464.9	464.9	-	-	-	307.7
NHLM-Z	-	403.2	-	-	-	-	333.5	333.5	333.5	552.7
CLM-R	241.9	-	241.9	451.6	451.6	451.6	-	-	-	348.3
CLM-Z	242.3	-	242.3	-	-	-	279.5	279.5	279.5	509.0

**Table 2:** Chemical composition of base materials (wt.%) obtained by XRF.

Material	Si	Al	Fe	Ca	Mg	K	Ti	S
Lime hydrate	0.1	0.9	0.1	69.5	0.5	-	-	-
NHL	3.1	2.0	1.7	60.3	1.2	0.4	0.1	-
Portland cement	7.7	2.4	2.4	46.7	0.9	0.7	0.2	2.8
Quartz sand	45.0	1.7	-	-	0.2	-	0.1	-
Zeolite	34.7	8.9	0.8	1.6	1.1	2.6	0.1	-

**Table 3:** Elemental composition of zeolite fractions obtained using EDS.

Element (wt.%)	Fractions of Zeolite (mm)		
	1–2	0.5–1	0.063–0.5
O	50.6	50.6	52.7
Si	36.5	36.5	34.9
Al	6.4	6.4	6.3
K	3.2	3.3	3.0
Ca	2.5	2.4	2.1
Fe	0.7	0.8	1.1

**Table 4:** The macrostructural properties of the hardened renders.

Material	*ρ*_b_ (kg·m^−3^)	*ρ*_b_ (kg·m^−3^)	*ρ*_s_ (kg·m^−3^)	*ρ*_s_ (kg·m^−3^)	*Ψ* *(%)*	*Ψ* *(%)*
	28 Days	90 Days	28 Days	90 Days	28 Days	90 Days
LM-R	1756 ± 25	1783 ± 25	2598 ± 31	2612 ± 31	32.4 ± 0.6	31.7 ± 0.6
LM-Z	1141 ± 16	1156 ± 16	2274 ± 27	2237 ± 27	49.8 ± 1.0	48.3 ± 1.0
NHLM-R	1761 ± 29	1813 ± 25	2597 ± 31	2625 ± 32	32.2 ± 0.6	30.9 ± 0.6
NHLM-Z	1158 ± 16	1184 ± 17	2126 ± 26	2119 ± 25	45.5 ± 0.9	44.1 ± 0.9
CLM-R	1814 ± 25	1845 ± 26	2525 ± 30	2535 ± 30	28.1 ± 0.6	27.2 ± 0.5
CLM-Z	1226 ± 17	1234 ± 17	2116 ± 25	2096 ± 25	42.0 ± 0.8	41.1 ± 0.8

**Table 5:** The mechanical properties of the hardened renders.

Material	*f*_f_ (MPa)	*f*_f_ (MPa)	*f*_c_ (MPa)	*f*_c_ (MPa)	*E*_d_ (GPa)	*E*_d_ (GPa)
	28 Days	90 Days	28 Days	90 Days	28 Days	90 Days
LM-R	1.1	1.5	1.3	1.9	4.4	4.8
LM-Z	0.7	0.8	1.1	1.7	3.4	3.6
NHLM-R	1.1	1.8	4.3	5.5	4.1	5.4
NHLM-Z	0.9	1.1	2.1	3.7	3.6	4.9
CLM-R	2.7	2.8	8.1	9.1	10.9	11.2
CLM-Z	1.7	1.9	5.1	5.6	4.2	4.7

The expanded combined uncertainty of the measured parameters is not presented because of its small values.

**Table 6:** The water vapor resistance factor of the hardened plasters.

Material	Dry-cup		Wet-cup	
	*µ* (-)			
	28 Days	90 Days	28 Days	90 Days
LM-R	11.1 ± 0.3	10.9 ± 0.3	10.9 ± 0.3	10.6 ± 0.3
LM-Z	9.7 ± 0.3	9.4 ± 0.3	9.3 ± 0.3	8.6 ± 0.2
NHLM-R	12.4 ± 0.3	11.3 ± 0.3	10.7 ± 0.3	9.5 ± 0.3
NHLM-Z	11.4 ± 0.3	11.1 ± 0.3	9.6 ± 0.3	9.8 ± 0.3
CLM-R	23.3 ± 0.7	18.9 ± 0.5	21.0 ± 0.6	19.8 ± 0.6
CLM-Z	19.5 ± 0.5	18.2 ± 0.5	19.6 ± 0.5	19.3 ± 0.5

**Table 7:** Water transport properties of the hardened plasters.

Material	*A*_w_ (kg·m^−2^·s^−1/2^)		*κ*_app_ (m^2^·s^−1^)	
	28 Days	90 Days	28 Days	90 Days
LM-R	0.28	0.25	7.54 × 10^−7^	6.12 × 10^−7^
LM-Z	0.36	0.34	2.08 × 10^−6^	1.96 × 10^−6^
NHLM-R	0.28	0.23	8.34 × 10^−7^	4.98 × 10^−7^
NHLM-Z	0.33	0.32	2.30 × 10^−6^	2.17 × 10^−6^
CLM-R	0.13	0.12	4.02 × 10^−7^	3.78 × 10^−7^
CLM-Z	0.24	0.22	5.61 × 10^−7^	4.76 × 10^−7^

**Table 8:** Mass change (wt.%) and difference in porosity (absolute%) after salt crystallization test.

Material	Mass Change		Difference in Porosity	
	NaCl	Na_2_SO_4_	NaCl	Na_2_SO_4_
LM-R	−0.34	0.50	−1.1	−1.4
LM-Z	−0.18	1.12	2.3	1.6
NHLM-R	1.13	1.52	−2.2	−1.7
NHLM-Z	1.70	1.90	−1.1	−0.8
CLM-R	0.70	1.03	1.1	1.9
CLM-Z	1.04	2.13	0.5	0.4

## Data Availability

The data presented in this study are available on request from the corresponding author. The data are not publicly available due to privacy.

## References

[1] Chu V., Regev L., Weiner S., Boaretto E. (2008). Differentiating between anthropogenic calcite in plaster, ash and natural calcite using infrared spectroscopy: Implications in archaeology. J. Archaeol. Sci..

[2] Veiga M.R., Silva A.S., Tavares M., Santos A.R., Lampreia N. (2013). Characterization of renders and plasters from a 16th century portuguese military structure: Chronology and durability. Restor. Build. Monum..

[3] Veiga M.R., Fragata A., Tavares M., Magalhães A.C., Ferreira N. (2009). Inglesinhos convent: Compatible renders and other measures to mitigate water capillary rising problems. J. Build. Apprais.

[4] Maravelaki-Kalaitzaki P., Bakolas A., Moropoulou A. (2003). Physico-chemical study of Cretan ancient mortars. Cem. Concr. Res..

[5] Silva A.S., Borsoi G., Veiga M.R., Fragata A., Tavares M., Llera F., Barreiros B., Teixeira T., Válek J., Hughes J. (2012). Diagnosis, characterization and restoration of the internal renders of Santíssimo Sacramento church in Lisbon. Historic Mortars: Characterisation, Assessment and Repair. RILEM Bookseries.

[6] Papayianni I., Stefanidou M. (2007). Durability aspects of ancient mortars of the archeological site of Olynthos. J. Cult. Herit..

[7] Hughes J.J., Van Balen K., Bicer-Simsir B., Binda L., Elsen J., van Hees R., von Konow T., Lindqvist J.E., Maurenbrecher P., Papayanni I. (2012). RILEM TC 203-RHM: Repair mortars for historic masonry. Mater. Struct..

[8] Loureiro A.M.S., Paz S.P.A., Veiga M.R., Angélica R.S. (2020). Assessment of compatibility between historic mortars and lime-METAKAOLIN restoration mortars made from amazon industrial waste. Appl. Clay Sci..

[9] Callebaut K., Elsen J., van Balen K., Viaene W. (2001). Nineteenth century hydraulic restoration mortars in the Saint Michael’s Church (Leuven, Belgium): Natural hydraulic lime or cement?. Cem. Concr. Res..

[10] Giosuè C., Pierpaoli M., Mobili A., Ruello M.L., Tittarelli F. (2020). Multifunctional lightweight mortars for indoor applications to improve comfort and health of occupants: Thermal properties and photocatalytic efficiency. Front. Mater..

[11] Amanatidis G. European Policies on Climate and Energy Towards 2020, 2030 and 2050. https://www.europarl.europa.eu/RegData/etudes/BRIE/2019/631047/IPOL_BRI(2019)631047_EN.pdf.

[12] Directive 2010/31/EU of the European Parliament and of the Council of 19 May 2010 on the Energy Performance of Buildings. http://data.europa.eu/eli/dir/2010/31/oj.

[13] Fleiter T., Steinbach J., Ragwitz M., Arens M., Aydemir A., Elsland R., Fleiter T., Frassine C., Herbst A., Hirze S. Mapping and Analyses of the Current and Future (2020–2030) Heating/Cooling Fuel Deployment (Fossil/Renewables): Executive Summary. https://ec.europa.eu/energy/sites/default/files/documents/mapping-hc-excecutivesummary.pdf.

[14] Barbero-Barrera M.M., González F.J.N., Ramos L.M., García Santos A., van Balen K., Válek J., Groot C., Hughes J.J. (2010). Energy Renovation by Lime Renders. Historic Mortars and RILEM TC 203-RHM Final Workshop HMC2010. Proceedings of the 2nd Historic Mortars Conference HMC2010 and RILEM TC 203-RHM Final Workshop.

[15] Kolokotsa D., Maravelaki-Kalaitzaki P., Papantoniou S., Vangeloglou E., Saliari M., Karlessi T., Santamouris M. (2012). Development and analysis of mineral based coatings for buildings and urban structures. Sol. Energy.

[16] van Hees R., Veiga R., Slížková Z. (2017). Consolidation of renders and plasters. Mater. Struct..

[17] (2005). Sanierputzsysteme, Wissenschaftlich-Technische Arbeitsgemeinschaft für Bauwerkserhaltung und Denkmalpflege e.V..

[18] Barnat-Hunek D., Siddique R., Klimek B., Franus M. (2017). The use of zeolite, lightweight aggregate and boiler slag in restoration renders. Constr. Build. Mater..

[19] Pavlík Z.M., Pavlíková M.L., Balík L., Černý R. (2015). In-situ analysis of hygric performance of piaristic monastery building. AIP Conf. Proc..

[20] Groot C.R., van Hees R., T. Wijffels T. (2009). Selection of plasters and renders for salt laden masonry substrates. Constr. Build. Mater..

[21] Petkovic J., Huinink H.P., Pel L., Kopinga K., van Hees R.P.J. (2010). Moisture and salt transport in three-layer plaster/substrate systems. Constr. Build. Mater..

[22] Gonçalves T.D., Pel L., Delgado Rodrigues J. Worsening of dampness and salt damage after restoration interventions: Use of water-repellent additives in plasters and renders. Proceedings of the 1st Historical Mortars Conference (HMC08).

[23] Fragata A., Veiga M.R., Velosa A.L., Válek J., Groot C., Hughes J.J. (2010). Salt Crystallization in Substitution Renderd for Historical Constructions. Historic Mortars and RILEM TC 203-RHM Final Workshop HMC2010. Proceedings of the 2nd Historic Mortars Conference HMC2010 and RILEM TC 203-RHM Final Workshop.

[24] Lubelli B., Nijland T.G., van Hees R.P.J. (2011). Self-healing of lime based mortars: Microscopy observations on case studies. Heron.

[25] de Freitas V.P., Gonçalves P.F. (2008). Specification and time required for the application of a lime-based render inside historic buildings. Conserv. Patrim..

[26] Veiga R. (2017). Air lime mortars: What else do we need to know to apply them in conservation and rehabilitation interventions? A review. Constr. Build. Mater..

[27] Nogueira R., Pinto A.P.F., Gomes A. (2018). Design and behavior of traditional lime-based plasters and renders. Review and critical appraisal of strengths and weaknesses. Cem. Concr. Compos..

[28] Gulbe L., Vitina I., Setina J. (2017). The influence of cement on properties of lime mortars. Procedia Eng..

[29] Pacheco-Torgal F., Faria J., Jalali S. (2012). Some considerations about the use of lime–cement mortars for building conservation purposes in Portugal: A reprehensible option or a lesser evil?. Constr. Build. Mater..

[30] Vyšvařil M., Pavlíková M., Záleská M., Pivák A., Žižlavský T., Rovnaníková P., Bayer P., Pavlík Z. (2020). Non-hydrophobized perlite renders for repair and thermal insulation purposes: Influence of different binders on their properties and durability. Constr. Build. Mater..

[31] Arizzi A., Viles H., Cultrone G. (2012). Experimental testing of the durability of lime-based mortars used for rendering historic buildings. Constr. Build. Mater..

[32] Andrejkovičová S., Velosa A.L., Ferraz E., Rocha F. (2014). Influence of clay minerals addition on mechanical properties of air lime–metakaolin mortars. Constr. Build. Mater..

[33] Santos A.R., Veiga M.R., Matias L., Silva A.S., De Brito J. (2018). Durability and compatibility of lime-based mortars: The effect of aggregates. Infrastructures.

[34] Pavlík Z., Pokorný J., Pavlíková M., Zemanová L., Záleská M., Vyšvařil M., Žižlavský T. (2019). Mortars with crushed lava granulate for repair of damp historical buildings. Materials.

[35] Beycan Tatanoğlu Ö., Kockal N.U. (2020). Utilization of pumice of Burdur region and zeolite of Bigadiç-Balıkesir region as fine aggregate in construction materials. Bull. Min. Res. Exp..

[36] Klimek B., Szulej J., Ogrodnik P. (2020). The effect of replacing sand with aggregate from sanitary ceramic waste on the durability of stucco mortars. Clean Technol. Envir..

[37] Abadou Y., Kettab R., Ghrieb A. (2018). Durability of a repaired dune sand mortar modified by ceramic waste. Eng. Struct. Technol..

[38] Styczeń J., Barnat-Hunek D., Panek R., Franus W. (2020). The microstructural and physical properties of renovation renders with clinoptilolite, Na-P1 and Na-X zeolites. Constr. Build. Mater..

[39] Sun X., Liu H., Tian Z., Ma Y., Wang Z., Fan H. (2021). Feasibility and economic evaluation of grouting materials containing binary and ternary industrial waste. Constr. Build. Mater..

[40] Ahmadi B., Shekarchi M. (2010). Use of natural zeolite as a supplementary cementitious material. Cem. Concr. Compos..

[41] Aškrabić M., Vyšvařil M., Zakić D., Savić A., Stevanović B. (2021). Effects of natural zeolite addition on the properties of lime putty-based rendering mortars. Constr. Build. Mater..

[42] Pavlík V., Užáková M. (2016). Effect of curing conditions on the properties of lime, lime–metakaolin and lime–zeolite mortars. Constr. Build. Mater..

[43] Lanas J., Alvarey-Galindo J. (2003). Masonry repair lime-based mortars: Factors affecting the mechanical behavior. Cem. Concr. Res..

[44] Horn K. (2011). Lime Rendering-Sustainable Heritage Report No. 1.

[45] Tenconi M., Karatasios I., Bala’awi F., Kilikoglou V. (2018). Technological and microstructural characterization of mortars and plasters from the Roman site of Qasr Azraq, in Jordan. J. Cult. Herit..

[46] Cazalla O., Rodriguez-Navarro C., Sebastian E., Cultrone G. (2000). Aging of lime putty: Effects on traditional lime mortar carbonation. J. Am. Ceram. Soc..

[47] Methods of Test for Mortar for Masonry (1999). Part 3: Determination of Consistence of Fresh Mortar (by Flow Table).

[48] (1999). Methods of Test for Mortar for Masonry—Part 10: Determination of Dry Bulk Density of Hardened Mortar.

[49] Záleská M., Pavlík Z., Čítek D., Jankovský O., Pavlíková M. (2019). Eco-friendly concrete with scrap-tyre-rubber-based aggregate—Properties and thermal stability. Constr. Build. Mater..

[50] (1999). Methods of Test for Mortar for Masonry—Part 11: Determination of Flexural and Compressive Strength of Hardened Mortar.

[51] (2002). Methods of Test for Mortar for Masonry-Part 18: Determination of Water Absorption Coefficient Due to Capillarity Action of Hardened Mortar.

[52] Feng C., Guimarães A.S., Ramos N., Sun L., Gawin D., Konca P., Hall C., Zhao J., Hirsch H., Grunewald J. (2020). Hygric properties of porous building materials (VI): A round robin campaign. Build. Environ..

[53] Lauermannová A.-M., Lojka M., Jankovský O., Faltysová I., Pavlíková M., Pivák A., Záleská M., Pavlík Z. (2021). High-performance magnesium oxychloride composites with silica sand and diatomite. J. Mater. Res. Technol..

[54] Kumaran M. (1999). Moisture diffusivity of building materials from water absorption measurements. J. Therm. Envelope Build. Sci..

[55] (2016). Hygrothermal Performance of Building Materials and Product Determination of Water Vapour Transmission Properties.

[56] Pavlík Z., Trník A., Keppert M., Pavlíková M., Žumár J., Černý R. (2014). Experimental investigation of the Properties of lime-based plaster-containing pcm for enhancing the heat-storage capacity of building envelopes. Int. J. Thermophys..

[57] (2020). Natural Stone Test Methods—Determination of Resistance to Salt Crystallization.

[58] Lubelli B., van Hees R.P.J., Nijland T.G., van Breugel K., Koenders A.E.B. (2014). Salt crystallization damage: How realistic are existing ageing tests?. Proceedings of the 1st International Conference on Ageing of Materials & Structures Delft University of Technology.

[59] Granneman S.J.C., Lubelli B., van Hees R.P.J. (2019). Effect of mixed in crystallization modifiers on the resistance of lime mortar against NaCl and Na_2_SO_4_ crystallization. Constr. Build. Mater..

[60] (2016). Specification for Mortar for Masonry—Part 1: Rendering and Plastering Mortar.

[61] Veiga M., Aguiar J., Silva A.S., Carvalho S.F., Lourenço P., Roca P. (2001). Methodologies for characterisation and repair of mortars of ancient buildings. Historical Constructions.

[62] Silva B.A., Ferreira Pinto A.P., Gomes A. (2015). Natural hydraulic lime versus cement for blended lime mortars for restoration works. Constr. Build. Mater..

[63] Moropoulou A., Bakolas A., Moundoulas P., Aggelakopoulou E., Anagnostopoulou S. (2005). Strength development and lime reaction in mortars for repairing historic masonries. Cem. Concr. Res..

[64] Faria-Rodrigues P., Henriques F.M.A. (2004). Current mortars in conservation: An overview. Restor. Build. Monum..

[65] Ventolà L., Vendrell M., Giraldez P., Merino L. (2011). Traditional organic additives improve lime mortars: New old materials for restoration and building natural stone fabrics. Constr. Build. Mater..

[66] Papayianni I. (2006). The longevity of old mortars. Appl. Phys. A.

[67] Papayianni I., Groot C. (2005). Design and Manufacture of Repair Mortars for Interventions on Monuments and Historical Buildings.

[68] Silva B.A., Ferreira Pinto A.P., Augusto G. (2014). Infleunce of natural hzdraulic lime content on the properties of aerial lime-based mortars. Constr. Build. Mater..

[69] Parcesepe E., De Masi R.F., Lima C., Mauro G.M., Pecce M.R., Maddaloni G. (2021). Assessmet of mechanical and thermal properties of hem-lime mortar. Materials.

[70] Roels S., Carmeliet J., Hens H., Adan O., Brocken H., Cerny R., Pavlik Z., Hall C., Kumaran K., Pel L. (2004). Interlaboratory comparison of hygric properties of porous building materials. J. Therm. Envel. Build. Sci..

[71] Pavlíková M., Zemanová L., Záleská M., Pokorný J., Lojka M., Jankovský O., Pavlík Z. (2019). Ternary blended binder for production of a novel type of lightweight repair mortar. Materials.

[72] Hens H.S.L.C. (2006). The vapor diffusion resistance and air permeance of masonry and roofing systems. Build. Environ..

[73] Darr J.P., Davis S.Q., Kohno Y., McKenna K., Morales P. (2014). Morphological effects on the hygroscopic properties of sodium chloride–sodium sulfate aerosols. J. Aerosol Sci..

[74] Martin S.T. (2000). Phase transitions of aqueous atmospheric particles. Chem. Rev..

[75] Biskos G., Malinowski A., Russll L.M., Busseck P.R., Martin S.T. (2006). Nanosize effect on the deliquescence and the efflorescence of sodium chloride particels. Aerosol Sci. Technol..

